# Efficacy analysis of 26 cases of ejaculatory duct obstruction treated by prostatic utricle neck endoscopy

**DOI:** 10.3389/fsurg.2022.1031739

**Published:** 2022-11-10

**Authors:** Kun-Long Lv, Wen-Gong Sun, Tian-Biao Zhang, Tao Zheng, Yong-Hao Nan, Yong-Fei Liu, Yi-Fan Zhou, Rui Wang

**Affiliations:** ^1^ Department of Andrology, The First Affiliated Hospital of Zhengzhou University, Zhengzhou, China; ^2^ Department of Urology, PLA 988 Hospital, Zhengzhou, China

**Keywords:** transurethral seminal vesiculoscopy, seminal vesicle, ejaculatory duct, male infertility, sperm

## Abstract

**Objective:**

To evaluate the safety and efficacy of transvesical incision in the treatment of ejaculatory duct obstruction.

**Methods:**

The clinical data of 26 male infertile patients with ejaculatory duct obstruction were retrospectively analysed at the First Affiliated Hospital of Zhengzhou University from June 2020 to August 2021. All patients were treated with seminal vesicle neck incision for ejaculatory duct obstruction. The general clinical characteristics, intraoperative conditions and postoperative effects on the patients were recorded, and the therapeutic effect was evaluated.

**Results:**

The ejaculatory duct was found through fenestration, and the seminal vesicle gland was smoothly entered in 25 patients (96.2%). Among them, 22 cases underwent bilateral endoscopy and three underwent unilateral endoscopy. Sperm appeared in 23 cases (88.5%) 3 months after surgery. The sperm concentration and motility postoperatively at 6 months were higher than that at 3 months postoperatively. No postoperative complications, such as epididymitis or retrograde ejaculation, occurred.

**Conclusion:**

Searching for the ejaculatory duct *via* the neck of the prostatic utricle, assisted by a low-energy holmium laser, is a new method for the treatment of ejaculatory duct obstruction. Microscopic vision is clear using this approach and the postoperative complications are few, which has high value for clinical application.

## Introduction

Ejaculatory duct obstruction is one of the most common types of obstructive azoospermia. Accounting for approximately 1%–5% of male infertility (1), it is a disease that has in recent years been treatable *via* surgery. Among the surgical methods used, seminal vesiculoscopy is the first choice of treatment. At present, this method is generally used in the treatment of ejaculatory duct obstruction through the natural channel of the ejaculatory duct opening or a rupture of the wall in the prostatic utricle. In current clinical practice, both methods are time-consuming and have a high failure rate (2–4). Finding a simpler entry method is key for improving the therapeutic effect of seminal vesiculoscopy, including obtaining a high success rate and causing no obvious clinical side effects. Since June 2020, the authors’ hospital has adopted the technique of burning the mucosa with a low-energy holmium laser next to the neck of the prostatic utricle and inserting the vesiculoscope through the ejaculatory duct orifice, which greatly improves the success rate of a seminal vesiculoscopy in the treatment of ejaculatory duct obstruction, without obvious clinical side effects. The report is as follows.

## Materials and methods

### Clinical data

The current paper presents a retrospective analysis of 26 ejaculatory duct obstructive male infertile patients aged 20–36 years old (average age, 26.5 ± 7.5) from June 2020 to August 2021 at the First Affiliated Hospital of Zhengzhou University Andrology Clinic. These patients had had the condition for 0.5–3 years, with an average of 1.5 ± 1.3 years. All patients underwent semen parameter analysis, scrotal ultrasound, rectal ultrasound, and sex hormone examinations. This study was approved by the ethics committee of the authors’ hospital (ethics number: 2022-KY-0382-001).

The inclusion criteria were as follows: (1) married patients with no children born in at least the preceding year; (2) no sperm observed in semen examination; single semen sample volume <2 ml, semen pH < 7.0, seminal fructose level negative more than three times by semen examination (diagnosed as azoospermia); (3) normal secondary sexual characteristics, normal size, volume, and texture of bilateral testes, palpable bilateral vas deferens, and no congenital reproductive abnormalities; (4) normal sex hormones; (5) conform to at least one item from the diagnostic criteria of ejaculatory duct obstruction as follows (5): (a) seminal vesicle expansion >1.5 cm; (b) ejaculatory duct dilatation diameter >2.3 mm; (c) a cyst near or off the midline of the verumontanum; (d) calcified stone formation in the verumontanum or ejaculatory duct; (6) the patient was informed about and agreed to participate in this study.

The exclusion criteria were as follows: (1) low testicular spermatogenesis; (2) an abnormal or narrow urethra; (3) the absence of seminal vesicle glands or vas deferens; (4) congenital malformations of the seminal vesicle glands; (5) coagulation dysfunction; (6) patients who had contraindications to surgery or who could not receive surgical treatment.

#### Equipment

An SRM-H3B model YAG laser treatment machine equipped with a 272 μm fibre and seminal vesiculoscope with a diameter of F4.6-6.4. Testicular volume was measured with a Prader orchidometer.

### Treatment methods

Following successful general anaesthesia, the lithotomy position was adopted; the posterior urethra was entered with a diameter F4.6-6.4 seminal vesiculoscope through the urethral orifice to observe whether the verumontanum and urethral mucosa were abnormal. The ejaculatory duct opening was searched for by pressurized water irrigation. We did not find abnormally wide ejaculatory duct openings at or in the area of the verumontanum in the 26 cases of patients with ejaculatory duct obstruction.

**Figure 1:**
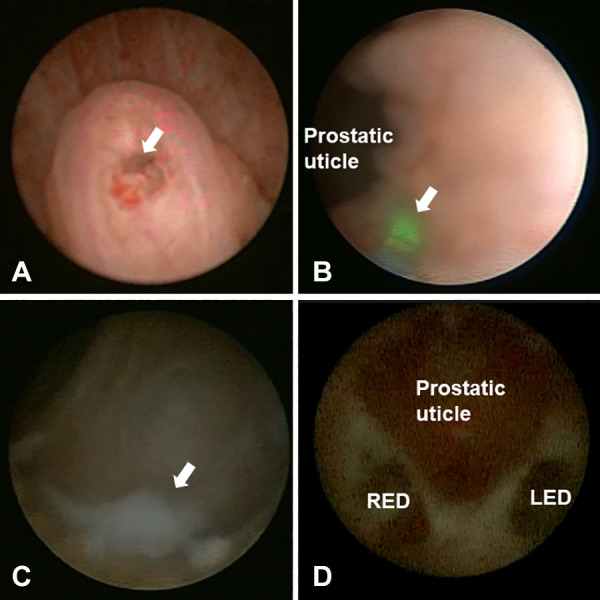
Intraoperative view of seminal vesiculoscopy in patients with ejaculatory duct obstruction. (**A**) The arrow shows the opening of verumontanum. (**B**) The arrow shows the low-energy holmium laser were used at five o'clock of the prostatic utricle neck to help find the ejaculatory duct opening. (**C**) The arrow shows white seminal fluid in the seminal vesicle. (**D**) The relationship between the prostatic utricle and the opening of ejaculatory duct. LED, left ejaculatory duct; RED, right ejaculatory duct.

Next, the doctor looked for the opening of the prostatic utricle at the verumontanum and entered the prostatic utricle through this opening. After removing the calculus in the small utricle, at an area with about 2–5 mm next to the neck of the prostatic utricle (at five o’clock on the left and seven o’clock on the right), a 272 μm thin optical fibre and a 2.0 HZ 1.2 J low-energy holmium laser were used. The villi were lightly cauterised at approximately 0.5–1.0 mm deep; then, a 20 ml syringe was used to pressurise water to rinse, find the opening of the ejaculatory duct and slowly enter the seminal vesicles through the ejaculatory duct opening. After entering the seminal vesicles, normal saline was used to rinse epeatedly until clear. In the case of stones, 2.0 HZ 1.2 J low-energy holmium laser lithotripsy was used to remove them. Finally, indwelling antibiotics were applied to the bilateral seminal vesicle gland, and the vesiculoscope was withdrawn. Figure 1 shows an example of a seminal vesiculoscopy in patients with ejaculatory duct obstruction.

### Postoperative treatment

The urinary catheter was removed 24–48 h after surgery, and antibiotics and haemostatic drugs were routinely used for 2 weeks. Two weeks after the operation, the patient was asked to ejaculate once a week. After surgery, routine semen analysis and outpatient physical examination took place every 3 months. Transrectal ultrasound, scrotum ultrasound, seminal plasma biochemistry and prostatic fluid analysis were reviewed every 3 months, and postoperative complications were recorded.

### Statistical processing

The SPSS Statistics 22.0 software was used to conduct analysis, and the measurement data were expressed as *x¯* ± *s*. The paired Wilcoxon test was used for comparison before and after the operation, and *P* < 0.05 indicated that the difference was statistically significant.

## Results

### General information

A total of 26 patients were enrolled, aged 20–36 (average age, 26.5 ± 7.5). They had had the disease for 0.5–3 years (average length 1.5 ± 1.3). In 26 patients, bilateral testes were 12–20 ml with a medium texture or above. The measured FSH value was 3.4–6.1 IU/L with a mean of 4.25 ± 1.97 IU/L. Among them, 10 patients had a reproductive history, and 5 other patients had sperm in the past. Additionally, 15 patients were diagnosed with prostatitis, 20 patients were diagnosed with epididymitis, 11 patients had a history of alcoholism, 6 patients were taxi drivers, and 5 other patients were engaged in long-term sedentary occupations.

### Introduction to the surgery

**Figure 2:**
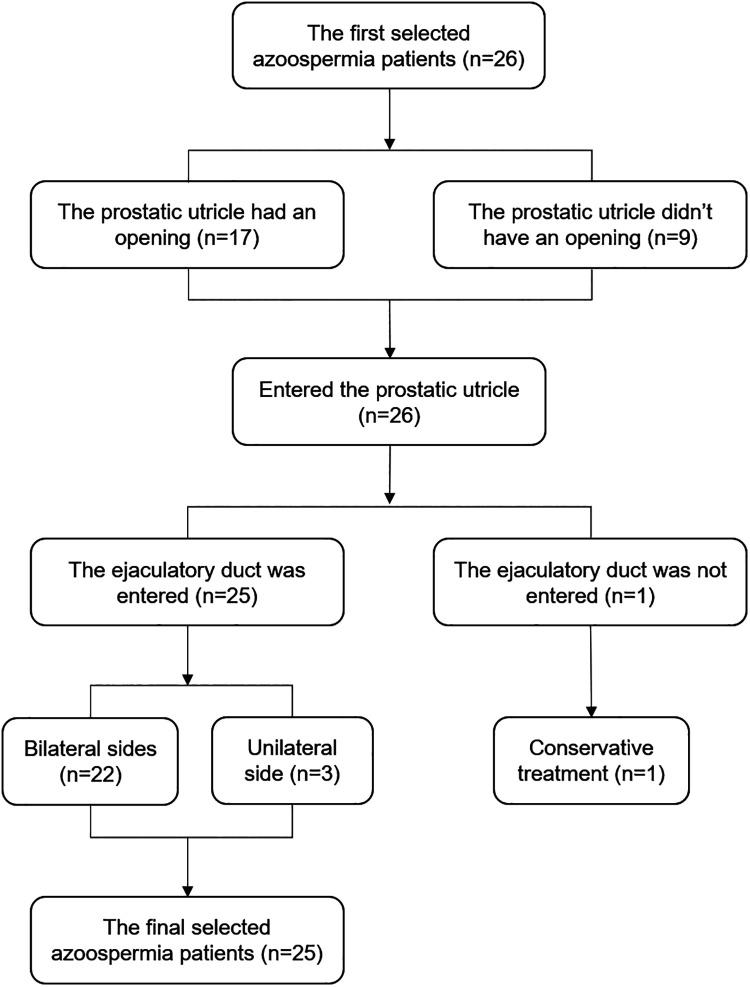
Flowchart of surgical procedure.

Of the 26 patients with ejaculatory duct obstruction, the prostatic utricle had an opening in 17 patients and other 9 didn’t have an opening. The seminal vesicle was entered smoothly through the ejaculatory duct in 25 patients. Twenty-two cases underwent bilateral ejaculatory duct endoscopy, and three cases underwent unilateral ejaculatory duct endoscopy (severe narrow opening of the ejaculatory duct on one side). In one case, the bilateral ejaculatory ducts were not found, and the patient was converted to receiving a conservative treatment. Stones were found in the seminal vesicle of four patients and the prostate of two patients. The surgical flow chart is shown in Figure 2.

### Sperm condition after surgery

Of the 25 successful seminal vesiculoscopy, 15 had sperm in the first month, 21 had sperm in the second month and 23 had sperm in the third month. Two patients were diagnosed with bilateral epididymal tail obstruction and no sperm was found 3 months after the surgery. These two patients underwent anastomosis of the bilateral vas deferens and epididymis under a microscope, and sperm could subsequently be observed in semen 3 months after the vasoepididymostomy. Two patients’ spouses were pregnant within 12 months after the vasoepididymostomy the surgery. Mild complications occurred in three patients, including epididymitis in one and haematuria in two cases. All of the patients had been cured after follow-up. None of the patients experienced severe complications (e.g., retrograde ejaculation, urinary incontinence or rectal injury).

### Comparison of seminal plasma biochemistry, semen parameters and seminal vesicle morphology before and 3 months after surgery

The semen volume after the surgery was significantly higher than before the operation (2.6 ± 1.8 vs. 0.5 ± 0.3), and the difference was statistically significant (*P* < 0.05). The postoperative semen pH value (7.3 ± 1.8) was significantly different compared with before the operation (6.0 ± 0.6) (*P* < 0.05). Postoperative semen fructose levels increased significantly (12.3 ± 6.8) compared with preoperative levels (0.8 ± 0.6), and the difference was statistically significant (*P* < 0.05). The postoperative neutral alpha-glucosidase (21.2 ± 12.8) increased compared with preoperatively (14.6 ± 8.2), and the difference was statistically significant (*P* = 0.038). The left length, left width, right length, right width of the seminal vesicle gland after surgery decreased compared with those before the operation, and the difference was statistically significant (*P* < 0.05). See Table 1 for details.

**Table 1:** Comparison of seminal plasma biochemistry and seminal vesicle gland morphology of 25 patients before and 3 months after operation.

Group	Preoperative	Postoperative	*P*
Semen volume (ml)	0.5 ± 0.3	2.6 ± 1.8	<0.001
PH	6.0 ± 0.6	7.3 ± 1.8	0.001
Fructose (umol)	0.8 ± 0.6	12.3 ± 6.8	<0.001
Neutral alpha-glucosidase (mU)	14.6 ± 8.2	21.2 ± 12.8	0.038
Seminal vesicle gland (mm)			
Left length	36.6 ± 2.8	24.7 ± 2.8	<0.001
Left width	15.9 ± 2.7	9.2 ± 2.7	<0.001
Right length	37.6 ± 4.6	23.3 ± 3.9	<0.001
Right width	15.8 ± 2.6	8.5 ± 2.3	<0.001

### Sperm concentration and sperm motility at 3 and 6 months after surgery

Six months after the surgery, the semen concentration of the patients increased compared with 3 months after the operation (20.4 ± 13.5 vs. 13.1 ± 10.2, *P* < 0.05). Sperm motility at 6 months after the surgery was significantly higher than at 3 months after the operation (43.9 ± 10.2 vs. 22.8 ± 8.3), and the difference was statistically significant (*P* < 0.05) as shown in Table 2.

**Table 2:** Comparison of sperm concentration and sperm motility of 25 patients at 3 and 6 months after operation.

	3 Months	6 Months	*P*
Concentration			
*N* (10^6^/ml)	13.1 ± 10.2	20.4 ± 13.5	0.037
A + B (%)	22.8 ± 8.3	43.9 ± 10.2	<0.001

A + B represents sperm motility grade A + B.

## Discussion

The classic treatment for ejaculatory duct obstruction is transurethral resection of ejaculatory ducts (6, 7). Although the therapeutic effect of this surgical method has been proven, it is more traumatic and more likely to have postoperative complications. In addition, some scholars have proposed the method of transrectal seminal vesicle imaging and balloon dilatation (8). This is an invasive procedure that is conducted under ultrasound guidance but is relatively less traumatic. However, few reports have been published on its use and the method's effect is not clear. The recently widely used procedure for ejaculatory duct obstruction (particularly in the case of complete obstruction) is seminal vesiculoscopy. Seminal vesicle fluid smear microscopy can be performed during surgery, with good diagnostic value and ejaculatory duct expansion and seminal vesicle cavity flushing can be performed. The relatively minimally invasive treatment of seminal vesiculoscopy can reduce complications and avoid surgical trauma while ensuring positive outcomes.

Seminal vesiculoscopy is currently the most direct and effective treatment for ejaculatory duct obstruction, but the success rate of entering the seminal vesicle is still low, which hinders its application. The two most commonly used approaches of seminal vesiculoscopy, through the natural opening of the ejaculatory duct or through the rupture of the prostatic sac, both have a high failure rate. According to existing reports (1–3), in the treatment of seminal vesiculitis, the clinical success rate of the first approach is only 1.9%, 2.3% and 3.2%, while for the latter this is only 80.14%, 75% and 88%. This is because the ejaculatory duct opening in normal anatomy is only 0.1–0.3 mm (9–11), which is much smaller than the tip of the 4.5F seminal vesiculoscope with a diameter of 1.5 mm. Even if the ejaculatory duct opening can be seen during surgery, the existing F4.5 seminal vesiculoscope will be difficult to insert.

Li et al. (12) found that the surface of the ejaculatory duct opening was covered with a layer of unidirectional villi, which concealed the ejaculatory duct opening. The opening of the ejaculatory duct is outside the 60° angle of the opening of the prostatic utricle, which makes it more difficult to insert the vesiculoscope through the natural cavity (13). The difficulty of penetrating the prostatic utricle is because only slightly more than half of prostatic utricles are larger than 5 mm. Prostatic utricles with a volume lower than 5 mm are considered small, and a small number of patients do not even have a prostatic utricle (14). At this time, the vertical distance between the bilateral ejaculatory ducts and the prostatic utricle is too large. Even if perfusion and aspiration are performed, it will not cause the walls of the prostatic utricle to vibrate. It is thus impossible to determine the exact location of puncture point in the small utricle during surgery, which increases the difficulty of breaking the wall of prostatic utricle, increasing the failure rate of the procedure.

The high success rate of breaking through the neck of the prostatic utricle into the seminal vesicle gland is due to the anatomical position of the neck of the prostatic utricle being relatively clear. Even if the opening of the prostatic utricle of some patients cannot be observed during surgery, the position of the neck can be determined after puncturing the prostatic utricle with a thin optical fibre. Additionally, the literature confirms that the positions of the bilateral ejaculatory ducts are located 2–3 mm beside the neck, at five and seven o’clock (11, 14, 15). Li et al. (12) examined the normal anatomy of a cadaver specimen and found that the horizontal distance from the vertex of the verumontanum to the left and right openings of the ejaculatory duct was 0.87 ± 0.10 mm, and the vertical distance was 1.36 ± 0.16 and 1.36 ± 0.15 mm, respectively, thus clearly indicating that the anatomical position of the bilateral ejaculatory duct opening is fixed.

A low-energy holmium laser burning at five and seven o’clock of the prostatic utricle neck can remove the unidirectional villi covering the opening of the ejaculatory duct, making it easy to reveal the opening of the ejaculatory duct during pressurised water injection. Moreover, clinical trials in the past year have also found that, after low-energy holmium laser burns, the success rate of finding bilateral ejaculatory ducts through the neck of the prostatic utricle is significantly higher compared with using a holmium laser to break through the prostatic utricle. Furthermore, the more obvious the villi, the faster the ejaculatory duct orifice can be identified, which is consistent with clinical findings presented by Shao et al. (13).

Whether cauterization of the ejaculatory duct opening with holmium laser will damage the ejaculatory duct, cause urine reflux, or re-stenosis of the ejaculatory duct, is a matter of concern. Recent studies have found that the 1.5cm-long ejaculatory duct is divided into three segments, the initial segment and the middle segment have a good muscular layer, while the end segment of the ejaculatory duct is only occasionally surrounded by a bundle of longitudinal muscle fibers, and there is no anti-reflux anatomical structure. Li (12) and others presented similar views. Based on current research, it is speculated that the anti-reflux mechanism of the ejaculatory duct is a result of the ejaculatory duct and seminal vesicle glands being in high-pressure conditions. The authors performed intraoperative hydrostatic pressure measurements on these two sites in 15 patients. The static pressure of the two sites was above 110 cm, and the hydrostatic pressure of the urethra was between 50 and 100 cm (16). The high hydrostatic pressure of the ejaculatory duct prevented urine from flowing back into the ejaculatory duct. Concurrently, this surgery essentially retains the 1.5 cm three-segment structure of the ejaculatory duct, which guarantees resistance to reflux. The results of 25 patients in this group at 3 and 6 months after surgery, and the absence of retrograde ejaculation and other side effects, confirmed the safety of this type of surgery.

Regarding the cause of ejaculatory duct obstruction, the first factor that was considered was inflammation. Twenty-five patients showed inflammatory changes in the ejaculatory ducts and seminal vesicles, and the rates of epididymitis and prostatitis were high. The rate of associated epididymitis and prostatitis was significantly reduced after operation. From the medical history, it can be seen that alcoholism, sedentary, ejaculatory duct cysts, and prostate cysts are the causes of inflammation.

There remains room for improvement in this surgical method. One of the 26 patients failed to find the opening of the ejaculatory ducts on both sides. Among the 25 successful cases, only one side of the ejaculatory duct was entered and the other ejaculatory duct could not be found. This indicated that variations remained in the relative position of the ejaculatory duct and the prostatic utricle. Accordingly, some experts use seminal vesicle gland massage to determine the position of the ejaculatory duct opening during surgery (13), which may help to improve the success rate of the surgery.

## Conclusion

Searching for the ejaculatory duct *via* the neck of the prostatic utricle with the assistance of a low-energy holmium laser is a new treatment method for ejaculatory duct obstruction. The microscopic vision is clear and the postoperative complications are few, which has high value for clinical application. Due to the small number of cases in this study, there were some limitations, i.e., not all patients were suitable for inclusion and the long-term effects still require evaluation by a large sample of controlled clinical studies.

## Data Availability

The original contributions presented in the study are included in the article/Supplementary Material, further inquiries can be directed to the corresponding author/s.
